# Obiltoxaximab Prevents Disseminated Bacillus anthracis Infection and Improves Survival during Pre- and Postexposure Prophylaxis in Animal Models of Inhalational Anthrax

**DOI:** 10.1128/AAC.01102-16

**Published:** 2016-09-23

**Authors:** Brent J. Yamamoto, Annette M. Shadiack, Sarah Carpenter, Daniel Sanford, Lisa N. Henning, Nestor Gonzales, Edward O'Connor, Leslie S. Casey, Natalya V. Serbina

**Affiliations:** a Elusys Therapeutics, Inc., Pine Brook, New Jersey, USA; b Battelle, West Jefferson, Ohio, USA

## Abstract

The Centers for Disease Control and Prevention recommend adjunctive antitoxins when systemic anthrax is suspected. Obiltoxaximab, a monoclonal antibody against protective antigen (PA), is approved for treatment of inhalational anthrax in combination with antibiotics and for prophylaxis when alternative therapies are not available. The impact of toxin neutralization with obiltoxaximab during pre- and postexposure prophylaxis was explored, and efficacy results that supported the prophylaxis indication are presented here. New Zealand White rabbits and cynomolgus macaques received obiltoxaximab as a single intramuscular or intravenous dose of 2 to 16 mg/kg of body weight at various times relative to Bacillus anthracis aerosol spore challenge. The primary endpoint was survival, and effect of treatment timing was explored. In rabbits, obiltoxaximab administration 9 h postchallenge singly or combined with a 5-day levofloxacin regimen protected 89% to 100% of animals compared to 33% with levofloxacin monotherapy. In cynomolgus macaques, a single intramuscular dose of 16 mg/kg obiltoxaximab led to 100% survival when given 1 to 3 days preexposure and 83% to 100% survival when given 18 to 24 h postexposure and prior to systemic bacteremia onset. Obiltoxaximab administration after bacteremia onset resulted in lower (25% to 50%) survival rates reflective of treatment setting. Prophylactic administration of obiltoxaximab before spore challenge or to spore-challenged animals before systemic bacterial dissemination is efficacious in promoting survival, ameliorating toxemia, and inhibiting bacterial spread to the periphery.

## INTRODUCTION

Anthrax is caused by the Gram-positive, spore-forming bacterium Bacillus anthracis, which is classified as a category A priority pathogen for biodefense. Inhalational anthrax can lead to fatality rates approximating 50% in humans, even when managed under optimal circumstances, and close to 100% if untreated (1, 2). There is a recognized need for effective treatment and prophylaxis strategies encompassing both standard antimicrobial and adjunctive components under scenarios of mass outbreak-exposure following intentional spore dispersal (3, 4), and antibody-based antitoxins are stockpiled by the U.S. government (4, 5). Current recommendations for postexposure prophylaxis (PEP) following anthrax spore exposure consist of an antibiotic regimen for immediate protection in conjunction with a 3-dose vaccine regimen (4, 5). While vaccination is projected to provide long-lasting protection, complete seroconversion requires at least 2 injections (6, 7). In addition, a monoclonal antibody, raxibacumab (8), is indicated for prophylaxis of inhalational anthrax when alternative therapies are not available or are not appropriate.

Obiltoxaximab (ETI-204), a chimeric IgG1(κ) monoclonal antibody, neutralizes protective antigen (PA) by preventing binding to cellular receptors (9, 10) and was recently approved for treatment of inhalational anthrax at a therapeutic dose of 16 mg/kg of body weight intravenously in combination with appropriate antibacterial drugs and for prophylaxis when alternative therapies are not appropriate or available under the U.S. Food and Drug Administration's (FDA's) Animal Rule (Code of Federal Regulations 21 CFR 601.90).

Studies in the New Zealand White (NZW) rabbit model of inhalation anthrax suggested that obiltoxaximab is protective when given at the time of exposure (11). Here, the protective efficacy of obiltoxaximab in the settings of both preexposure prophylaxis and postexposure prophylaxis (after exposure to the aerosolized spores but prior to the development of clinical symptoms) settings was explored in the NZW rabbit and cynomolgus macaque models of inhalational anthrax in studies designed to simulate human clinical trials. The goals of these studies were to (i) establish obiltoxaximab efficacy in the prophylaxis setting following intravenous (i.v.) and intramuscular (i.m.) administration; (ii) define the window of effectiveness for prophylaxis; and (iii) explore conditions where obiltoxaximab may provide added benefit to prophylactic antibiotic therapy.

## MATERIALS AND METHODS

### Test system.

NZW rabbits (Oryctolagus cuniculus, specific pathogen free), weighing 2.2 to 2.7 kg, were procured from Covance Research Products, Inc. (Denver, PA).

Cynomolgus macaques (Macaca fascicularis) weighing 2.1 to 5.6 kg were procured from Covance, Inc. (Alice, TX). Macaques were verified negative for tuberculosis and prescreened within 30 days prior to receipt at Battelle Biomedical Research Center, Columbus, Ohio, confirming they were seronegative for simian immunodeficiency virus, simian T-lymphotrophic virus 1, and cercopithecine herpesvirus 1 and negative for simian retroviruses 1 and 2 by PCR. Only healthy macaques free of malformations and clinical signs of disease and negative for intestinal parasites were placed on study.

### Study designs.

All studies used a randomized, controlled, parallel-group (50% male, 50% female) design. Animals were challenged with aerosolized B. anthracis (Ames) spores at 200 times the median lethal dose (12, 13). Aerosol challenges were conducted as previously described (12, 14). Briefly, spores were aerosolized by a Collison nebulizer and delivered via a nose-only (rabbits) or head-only (macaques) inhalation exposure chamber. Aerosol concentrations of B. anthracis were quantified by collecting effluent stream samples directly from an animal exposure port by an in-line, all-glass impinger and plating serial dilutions of the impinger samples onto tryptic soy agar plates. Real-time plethysmography was performed to calculate the inhaled spore dose. In preexposure studies, cynomolgus macaques received a single i.m. dose of 16 mg/kg of body weight of obiltoxaximab or control (vehicle) in the thigh at 24, 48, or 72 h before spore challenge. In the PEP studies, a single dose of obiltoxaximab or control (saline or vehicle) was administered either by i.v. bolus or i.m. injection to rabbits or macaques at a range of times following spore challenge (9, 18, 24, 36, and 48 h).

Animals were randomized by sex and weight to treatment. Animals were then randomized to an aerosol challenge day and challenge order such that there were approximately equal numbers of animals from each group on each challenge day. Survival rate was the primary endpoint, defined as the proportion of animals alive at the time of scheduled study termination. Blinding was used in the preexposure study and one postexposure study (PEP 2).

### Study conduct.

Spore challenges were performed as described previously (14), and delivered spore doses were measured by real-time plethysmography. All studies were conducted at the biosafety level 3 facilities at Battelle Biomedical Research Center, Columbus, Ohio, with the approval of Battelle's Institutional Animal Care and Use Committee. All studies, with the exception of PEP 3, were conducted in compliance with the FDA's Good Laboratory Practice regulations (21 CFR Part 58). PEP 3 was conducted in accordance with Battelle standard operating procedures (SOPs), methods, and the study protocol. Deviations and investigations that could impact the study were recorded, included in the study report, and analyzed for potential impact, with the conclusion that none impacted the study.

### Treatment administration.

A single i.v. or i.m. obiltoxaximab dose, ranging from 2 to 16 mg/kg, was administered in sterile 0.9% sodium chloride for injection or in vehicle (40 mM l-histidine, 200 mM sorbitol, 0.01% polysorbate 80 [Tween 80]) alone or concurrently with the first of 5 daily oral doses of 50 mg/kg levofloxacin (rabbit only) or placebo (water). In the rabbit postexposure study, obiltoxaximab was given at a fixed dose of 10 or 20 mg/rabbit. The body weights of rabbits in this study ranged from 2.2 to 2.7 kg; thus, the actual doses administered were approximately 4 mg/kg i.v. and 8 mg/kg i.m. i.v. treatment was administered as a single bolus (via saphenous vein in the cynomolgus macaque study), and i.m. treatment was administered such that the maximum volume given per injection site (thigh) did not exceed 0.5 ml.

### PK and pharmacodynamic measurements.

In the preexposure study, blood samples for pharmacokinetic (PK) analyses were collected at 6 h posttreatment, at 24, 54, and 96 h postchallenge, and on days 7, 14, 28, and 56 postchallenge. Blood samples for PK analyses for study PEP 2 were collected at 1, 6, 12, 24, and 72 h posttreatment on days 7, 14, 21, 28, and 56 (16 mg/kg group only) postchallenge. Blood samples for PK analyses for study PEP 3 were collected at 12, 24, and 72 h posttreatment and on days 7, 14, and 28 postchallenge. A validated (15) enzyme-linked immunosorbent assay (ELISA) method was used to quantify obiltoxaximab in cynomolgus macaque serum. PA83 (List Biologicals, Campbell, CA) immobilized on microtiter plates was used as a capture reagent. Affinity-purified sheep antihuman IgG (monkey adsorbed; Binding Site, United Kingdom) conjugated to horseradish peroxidase (HRP) was used as the detection reagent with tetramethylbenzidine (BioFX, Owings Mills, MD) as the substrate. The lower limit of quantitation (LLOQ) of the assay was 100 ng/ml, and the upper limit of quantitation (ULOQ) was 5,000 ng/ml. PK parameters were based on composite mean concentrations from all animals per time point per dose group (male and female values combined), analyzed as 1 profile.

Samples for assessment of both PA and bacteremia were collected relative to median challenge time between 24 h postchallenge and time of treatment and between days 7 and 28 postchallenge. Subsequent to treatment and until day 7 postchallenge, samples were collected relative to treatment time for individual animals, at 24 and 72 h posttreatment. A validated (15, 16) ELISA method was used to quantify PA63 and/or PA83 in monkey serum using obiltoxaximab as the capture antibody and goat anti-PA antisera with HRP-conjugated anti-gamma chain secondary antibody as the reporter system. This assay had an LLOQ of 9.68 ng/ml and did not detect PA20 or PA bound to serum obiltoxaximab.

Bacteremia was quantified by culture of blood samples on blood agar plates at 37°C for 16 to 24 h. Fresh samples were serially diluted 1:10 and plated in triplicate. Dilutions were acceptable if the number of CFU fell between 25 and 250 CFU/plate. Bacterial concentration (in CFU/ml) was determined as [(mean number of colonies on 3 plates) × (total dilution factor)]/(inoculation volume, in milliters). For tissue bacteremia assessment, representative samples of the lymph nodes, spleen, liver, and brain were collected, homogenized, and plated on blood agar plates for qualitative assessment of bacterial presence.

### Statistical considerations and survival modeling.

Designs of pre- and postexposure studies are shown in Table 1. All power calculations were done prospectively. The sample size for the postexposure rabbit study was chosen to provide >80% power to detect an improvement in survival if survival was 10%, 30%, and 80% in the control, levofloxacin-only, and obiltoxaximab groups, respectively. The sample size for the preexposure prophylaxis macaque study was chosen to provide 80% power to detect an improvement in survival of 60% compared to the control group. The sample size for the macaque PEP studies was chosen to provide 80% power to detect an improvement in survival in each of the obiltoxaximab groups of 70% in study PEP 1 and 55% in study PEP 3. Sample size calculations were not performed for study PEP 2.

**TABLE 1:** Study overview

Study	Study design	Treatment regimen and/or dose	Total no. of animals	Mean LD_50_ challenge spore dose (SD)
NZW rabbits				
PEP	Postexposure, dose-ranging, and levofloxacin combination study in challenged animals; dose received 9 h after anthrax exposure	0 mg/kg i.v.	9	268.6 (47.5)
		4 mg/kg i.v.	9	287.8 (69.5)
		4 mg/kg i.v. + levofloxacin at 50 mg/kg/day p.o. for 5 days	9	262.3 (40.8)
		8 mg/kg i.m.	9	270.4 (38.4)
		8 mg/kg i.m. + levofloxacin at 50 mg/kg/day p.o. for 5 days	9	252.6 (41.7)
		levo at 50 mg/kg/day p.o. for 5 days	12	297.3 (55.2)
Cynomolgus macaques				
Preexposure	Prophylaxis; i.m. dose received within 24, 48, and 72 h preexposure	0 mg/kg (24, 48, 72 h)	10	217.8 (65.2)
		16 mg/kg (24 h)	14	237.3 (96.1)
		16 mg/kg (48 h)	14	209.3 (61.6)
		16 mg/kg (72 h)	15	220.2 (86.7)
PEP 1	Postexposure; i.v./i.m. dose-ranging study in challenged animals; dose received 24 h postexposure	0 mg/kg i.v.	6	324.2 (70.6)
		2 mg/kg i.v.	9	315.6 (83.4)
		8 mg/kg i.v.	9	288.7 (49.1)
		4 mg/kg i.m.	8	366.0 (113.56)
		8 mg/kg i.m.	9	289.0 (51.8)
PEP 2	Postexposure; i.m. dose-ranging study at increasing times postexposure (18–36 h)	0 mg/kg (18 h)	6	395.7 (166.8)
		8 mg/kg (18 h)	6	461.7 (151.6)
		16 mg/kg (18 h)	6	422.8 (157.8)
		8 mg/kg (24 h)	6	385.5 (133.4)
		16 mg/kg (24 h)	6	305.2 (130.2)
		8 mg/kg (36 h)	6	409.5 (131.1)
		16 mg/kg (36 h)	6	431.8 (215.4)
PEP 3	Postexposure; i.m. obiltoxaximab efficacy at increasing times postexposure (24–48 h)	0 mg/kg (24 h)	10	200.7 (45.9)
		16 mg/kg (24 h)	14	209.0 (56.8)
		16 mg/kg (36 h)	14	197.6 (92.4)
		16 mg/kg (48 h)	16	208.9 (67.8)

The survival rate in each of the obiltoxaximab groups was compared to that of the control using descriptive statistics and Boschloo's exact test with a Berger-Boos correction of gamma of 0.001 (17) for a modified intent-to-treat population. Modified intent-to-treat population was defined as all challenged animals in studies PEP 1 and PEP 3 and all challenged animals who received treatment in study PEP 2. Analyses were conducted with R using the package “exact.” Exact 95% confidence intervals (CI) for differences in survival rates are based on the score statistic (Proc Freq of S, SAS, version 9).

A Weibull cure-rate model (18, 19) was implemented to describe survival for the monkey efficacy data set. The survival model also included an *E*_max_ (maximal effect on *p*_surv_ on the logit scale) dose-response and an exponential effect of log_10_(PTT bacteremia) on logit(*p*_surv_), where *p*_surv_ represents the proportion of animals surviving and PTT is prior to treatment. Two covariates, dose (milligram per kilogram) and PTT quantitative bacteremia, were evaluated for the effect on the survival.

## RESULTS

### Preexposure prophylaxis with obiltoxaximab.

**FIG 1:**
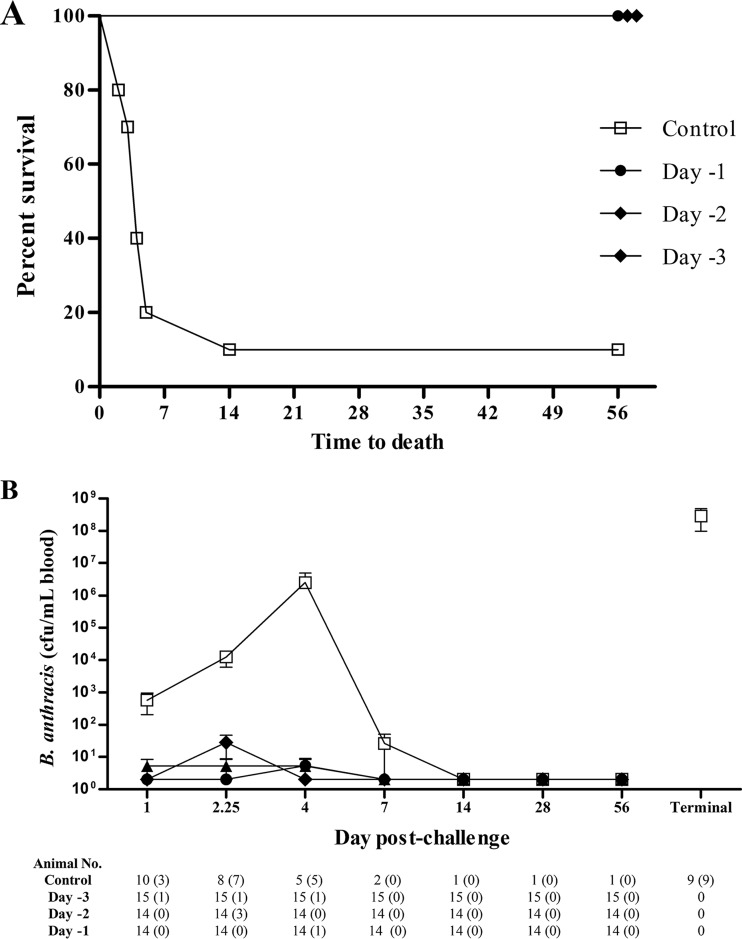
Preexposure prophylaxis with obiltoxaximab. Cynomolgus macaques were administered a single i.m. dose of obiltoxaximab or vehicle 1 to 3 days prior to aerosol challenge with targeted 200 LD_50_ of B. anthracis spores, and animals were monitored to 56 days postchallenge. (A) Kaplan-Meier curves representing time to death from challenge and survival data for each group are shown. (B) Peripheral blood samples were collected at the indicated times postchallenge for quantitative assessment of bacteremia from control animals (open squares) or animals that received obiltoxaximab 3 days (closed triangles), 2 days (closed diamonds), and 1 day (closed circles) prior to challenge. Terminal samples were collected at study termination for individual animals. Shown are means and standard deviations from each study. Numbers of animals surviving to each sample collection are indicated at the bottom and numbers in parentheses indicate numbers of animals positive for bacteremia. Bacteremia levels below the limit of detection (LOD) were replaced with 3 CFU/ml (1/2 LOD).

Administration of a single i.m. dose of 16 mg/kg obiltoxaximab to cynomolgus macaques 24, 48, and 72 h prior to lethal challenge with B. anthracis spores (Table 1) led to 100% survival in all treatment groups (14/14, 14/14, and 15/15, respectively), whereas only 10% (1/10) of control-treated animals survived (Fig. 1A). The only control group survivor received a challenge dose of 20,400,000 CFU (330 median [50%] lethal doses [LD_50_]). The impact of obiltoxaximab administration on development of blood bacteremia following spore challenge was examined. In controls, blood bacteremia generally increased exponentially between days 1 and 4 postchallenge with high levels in terminal samples. In the single control survivor, bacteremia resolved between days 4 and 7 (Fig. 1B). In obiltoxaximab-treated animals, transient low levels of bacteremia were observed in several animals, resolving in all animals after day 4, and the majority of animals remained abacteremic until the end of the study (day 56 postchallenge) (Fig. 1B). Bacteria could not be cultured from any peripheral tissue examined (the lymph nodes, spleen, liver, or brain) of any of the surviving animals but was cultured from all tissues in 8 of 9 control nonsurvivors.

### Obiltoxaximab efficacy in the PEP setting.

**FIG 2:**
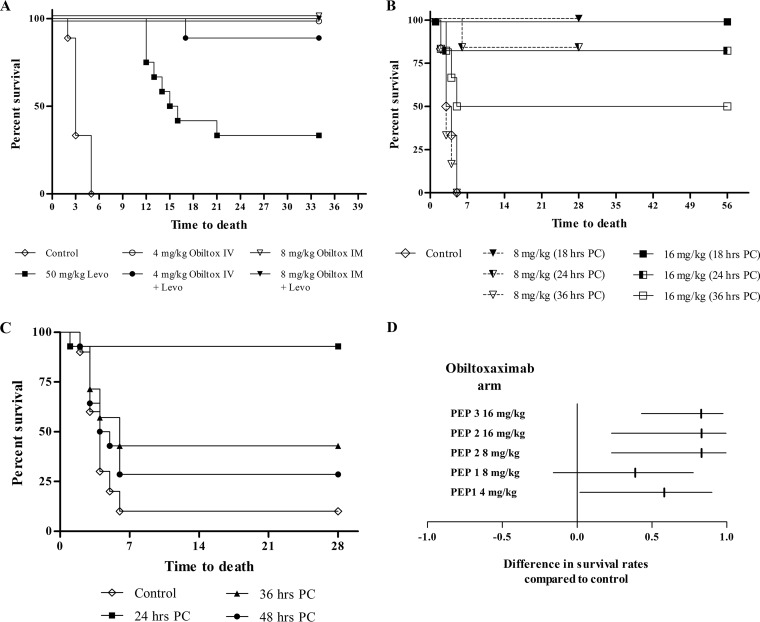
Postexposure prophylaxis with obiltoxaximab. (A) NZW rabbits were aerosol challenged with targeted 200 LD_50_ of B. anthracis spores, and obiltoxaximab was administered 9 h postchallenge either alone or in combination with the first of 5 daily oral doses of 50 mg/kg levofloxacin or levofloxacin alone. Kaplan-Meier curves representing time to death from challenge and survival data for each group are shown. (B to D) Three cynomolgus macaque postexposure prophylaxis studies were conducted. Animals were challenged with targeted 200 LD_50_ of B. anthracis spores, and a single dose of 4 to 16 mg/kg obiltoxaximab was administered intramuscularly at various times postchallenge. Animals were monitored for 28 or 56 days. Control animals received placebo injection 24 h (study PEP 1) or vehicle injection 18 h (study PEP 2) and 24 h (study PEP 3) postchallenge. (B and C) Kaplan-Meier curves representing time to death from challenge and survival data for each group in study PEP 2 (B) and study PEP 3 (C). (D) Forest plot of survival results for groups treated at 24 h postchallenge in studies PEP 1 to 3. The forest plot represents differences between survival proportions in placebo and obiltoxaximab treatment groups (vertical lines) with corresponding confidence intervals (horizontal lines).

A series of studies were conducted to investigate obiltoxaximab effectiveness in postexposure prophylaxis. The design of these studies was informed by the FDA guidance that defines prophylaxis as a window of opportunity for preventing illness and reducing mortality after exposure to spores but prior to development of signs and symptoms of inhalational anthrax (20). Obiltoxaximab effectiveness in the PEP setting was initially examined in rabbits. Administration of obiltoxaximab at doses of 4 and 8 mg/kg (i.v. and i.m., respectively) 9 h following lethal spore challenge led to 100% survival compared to 0% in the saline control group (*P* = 0.001 for each group). The survival rate was 33.3% in rabbits that received 50 mg/kg levofloxacin for 5 days with subsequent discontinuation (*P* = 0.048 compared to the control group) (Fig. 2A). Survival rates were 89% and 100% in rabbits treated with the combination of levofloxacin and 4 mg/kg i.v. or 8 mg/kg i.m. obiltoxaximab, respectively (*P* = 0.001 for each group) (Fig. 2A).

In cynomolgus macaques, bacteria disseminates to blood approximately 12 h after transport to thoracic lymph nodes (21) and approximately 37 h after spore challenge, marking the beginning of systemic infection (14). In 3 studies, a single dose of 2 to 16 mg/kg obiltoxaximab was administered to cynomolgus macaques i.v. or i.m. 18 to 48 h following spore challenge (Table 1). In study PEP 1, administration of 2 and 4 mg/kg obiltoxaximab i.v. led to survival rates of 44% (4/9) and 67% (6/9), respectively (data not shown), and administration of 4 and 8 mg/kg obiltoxaximab i.m. led to 75% (6/8) and 56% (5/9) survival compared to 17% (1/6) survival in the control group (Fig. 2D). In study PEP 2, 8 or 16 mg/kg obiltoxaximab resulted in survival of all animals when administered at 18 h and 83% survival when administered at 24 h following challenge, compared to 0% control survival (Fig. 2B and Table 2). When obiltoxaximab was given at 36 h postchallenge, the survival rate was reduced to 0% and 50% in the 8 mg/kg and 16 mg/kg groups, respectively (Fig. 2B and Table 2). To further define the prophylactic treatment window, study PEP 3 was conducted with 16 mg/kg obiltoxaximab administered i.m. at 24, 36, and 48 h following spore challenge (Table 1). Administration of obiltoxaximab at 24 h postchallenge led to 92.9% survival compared to 10% in the control group; survival was reduced when obiltoxaximab was given at 36 or 48 h (Fig. 2C and Table 2). In all 3 studies, obiltoxaximab administration at 24 h following spore challenge led to an improvement in the survival rate compared to that of the control (Fig. 2D). In 2 control survivors in studies PEP 1 and PEP 3, administered spore doses were 17,600,000 CFU (285 LD_50_) and 16,400,000 CFU (265 LD_50_), suggesting that survival in the absence of treatment was not due to aerosolization failure.

**TABLE 2:** Survival results and bacteremia status in cynomolgus macaque PEP studies

Dose group (mg/kg)	Treatment time (h)	PEP 2 (study end, 28–56 days^*a*^)					PEP 3 (study end, 28 days)				
		Proportion surviving (no. [%])	*P* value^*d*^	95% CI	Proportion bacteremic^*b*^ (no. [%])	Bacteremia^*c*^ (mean, [SD])	Proportion surviving (no. [%])	*P* value^*d*^	95% CI	Proportion bacteremic^*b*^ (no. [%])	Bacteremia^*c*^ (mean, [SD])
Placebo		0/6 (0)			0/6 (0)	NA	1/10 (10)			2/10 (20)	1.6 × 10^2^ (1.5 × 10^2^)
8	18	6/6 (100)	0.0012*	0.471, 1.000	0/6 (0)	NA					
16	18	6/6 (100)	0.0012*	0.471, 1.000	0/6 (0)	NA					
8	24	5/6 (83)	0.0042*	0.230, 0.996	1/6 (16.7)	5.4 × 10^3^					
16	24	5/6 (83)	0.0042*	0.230, 0.996	1/6 (16.7)	7.1 × 10^3^	13/14 (93)	0.001*	0.431, 0.976	1/14 (7.1)	6.0 × 10^2^
8	36	0/6 (0)	1.0000		6/6 (100)	9.2 × 10^4^ (8.8 × 10^4^)					
16	36	3/6 (50)	0.0345	−0.037, 0.882	5/6 (83.3)	1.2 × 10^7^ (2.7 × 10^7^)	6/14 (43)	0.0536	−0.068, 0.643	9/14 (64.3)	1.3 × 10^6^ (2.6 × 10^6^)
16	48						4/16 (25)	0.2196	−0.214, 0.454	16/16 (100)	7.7 × 10^6^ (2.3 × 10^7^)

a Animals in the 8-mg/kg group were sacrificed 28 days postchallenge, and animals in the 16-mg/kg group were sacrificed 56 days postchallenge.

b Animals positive by quantitative bacteremia culture at PTT.

c Only animals positive for bacteremia prior to treatment are included; results above the limit of detection but below the limit of quantitation were assigned a value of 1/2 LOD. NA, not applicable.

d An asterisk denotes statistical significance at the 0.025 level.

### Treatment efficacy by bacteremia status.

**FIG 3:**
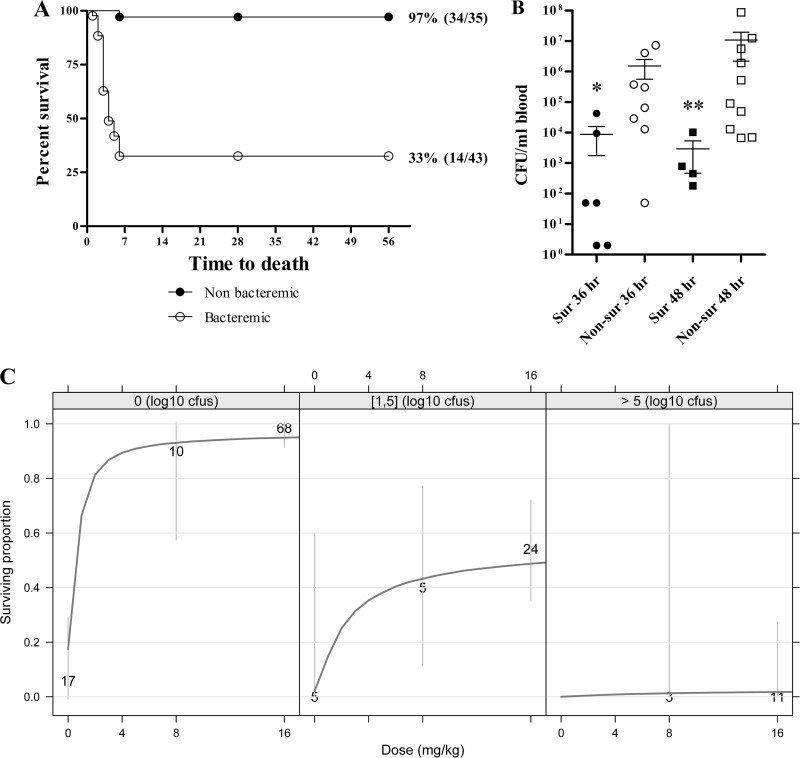
Impact of pretreatment bacteremia on survival outcomes. (A) Survival data from studies PEP 2 and PEP 3 were integrated to examine the impact of bacteremia status on treatment efficacy. Shown are Kaplan-Meier curves representing time to death from challenge and survival data for animals that were nonbacteremic (closed circles) and bacteremic (open circles) across all treatment doses and times. Control animals were not included. (B) Levels of circulating B. anthracis in study PEP 3 were assessed quantitatively immediately prior to treatment. Shown are geometric means with corresponding 95% confidence intervals for surviving (Sur) and nonsurviving (Non-Sur) animals that received treatment at 36 and 48 h postchallenge. For statistical computations, bacteremia levels below the LOD were replaced with 2 CFU/ml (1/2 LOD), and levels below limits of quantitation were replaced with 50 CFU/ml (1/2 LOQ). Statistical significance was compared in survivors and nonsurvivors by Mann-Whitney test with GraphPad Prism 5. (C) Dose-response model of survival stratified by level of prior-to-treatment (PTT) bacteremia. Data are pooled across studies Preexp, PEP 2, and PEP 3. The 3 plots represent different ranges of PTT bacteremia observed across the studies. Dark grey lines represent the model predictions. Gray vertical lines represent 95% Agresti-Couli confidence intervals. Numbers on dark grey lines indicate the total number of animals comprising each dose group.

Transition to systemic disease was assessed by measuring blood bacteremia immediately prior to treatment in studies PEP 2 and PEP 3. All animals were negative for bacteremia at 18 h postchallenge in study PEP 2, and 16.7% and 7.1% of animals had bacteria in circulation at 24 h postchallenge in studies PEP 2 and PEP 3, respectively (Table 2). Across both studies, 65% of animals had evidence of a systemic infection by 36 h following spore challenge, and all animals in study PEP 3 were bacteremic at 48 h postchallenge (Table 2). Survival in studies PEP 2 and PEP 3 was then analyzed by stratifying animals by bacteremia status. Obiltoxaximab treatment prior to bacterial dissemination to the periphery resulted in 93.7% survival irrespective of time of administration; survival was lower (36.1%) when obiltoxaximab was administered i.m. after establishment of systemic disease (Fig. 3A).

Correlation between levels of bacteremia at treatment and survival outcomes in individual animals was explored. In study PEP 3, pretreatment bacteremia at either 36 or 48 h postchallenge was significantly higher in nonsurviving animals than surviving animals (Fig. 3B). Modeling was conducted to describe survival across all macaque pre- and postexposure studies and to examine whether pretreatment bacteremia levels in animals were prognostic for survival. As shown in Fig. 3C, near-maximal survival was predicted with i.m. obiltoxaximab doses of 8 and 16 mg/kg for animals with no detectable blood bacteremia prior to treatment. When pretreatment bacteremia levels were <10^5^ CFU/ml, an obiltoxaximab dose of 16 mg/kg provided a small additional survival benefit relative to 8 mg/kg. A very low probability of survival was predicted for obiltoxaximab monotherapy when pretreatment bacteremia levels were >10^5^ CFU/ml (Fig. 3C).

### Systemic disease after obiltoxaximab prophylaxis.

**FIG 4:**
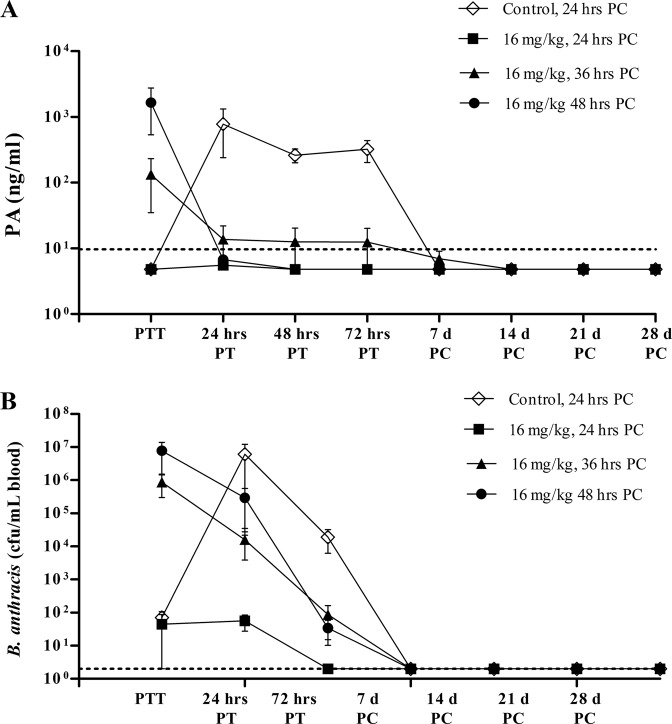
Development of bacteremia and toxemia during postexposure prophylaxis (PEP). Cynomolgus macaques in study PEP 3 were aerosol challenged with targeted 200 LD_50_ of B. anthracis spores, and obiltoxaximab was administered at 24, 36, and 48 h postchallenge. Control animals received vehicle at 24 h postchallenge. Peripheral blood samples were collected immediately prior to treatment (PTT) or at indicated times posttreatment (PT) or postchallenge (PC) for the assessment of circulating free PA (A) and quantitative bacteremia (B). Shown are means and standard errors of the means for free PA and bacteremia at each indicated time point. Dotted lines represent LOQ (PA) and LOD (bacteremia). Numbers of animals surviving to each sample collection are indicated at the bottom. For statistical computations, PA levels below the LOQ were replaced with 4.84 ng/ml (1/2 LOQ), and bacteremia levels below the LOD and LOQ were replaced with 2 CFU/ml (1/2 LOD) and 50 CFU/ml (1/2 LOQ), respectively. The control group was comprised of only one survivor starting at day 7.

Bacteremia and toxemia development following administration of obiltoxaximab were examined in studies PEP 2 and PEP 3. Animals treated at 18 and 24 h postchallenge did not have detectable free serum PA at the time of treatment and remained free of circulating PA throughout the study (Fig. 4A and data not shown). Complete and irreversible elimination of PA was seen in animals treated at 36 and 48 h postchallenge (Fig. 4A and data not shown). As expected, PA levels rose exponentially during the first 24 h posttreatment in animals administered vehicle 24 h postchallenge.

The majority of macaques were not bacteremic at 18 and 24 h postchallenge (Table 2), and administration of 16 mg/kg obiltoxaximab at these times prevented systemic bacteremia in the vast majority of animals, with low and transient levels observed in a few animals only (Fig. 4B and data not shown). Obiltoxaximab administration at 36 and 48 h following spore challenge, when the majority of animals were bacteremic, led to a reduction in bacterial levels as early as 24 h posttreatment and complete resolution of bacteremia in all surviving animals by day 7. In contrast, bacteremia levels rose exponentially in the control group administered vehicle 24 h postchallenge during the first 24 h following treatment with very high mean bacteremia in terminal samples in study PEP 3 (1.4 × 10^8^; standard deviations, 5.8 × 10^7^ CFU/ml). While the study did not include separate control groups to match groups treated with obiltoxaximab at 36 and 48 h postchallenge, the course of bacteremia and PA kinetics in the PEP 3 control group were comparable to those observed in control groups of treatment studies when saline is administered after first symptoms of systemic disease (34, and data not shown). Thus, levels of bacteremia and PA in the PEP 3 control group provide a useful baseline to gauge the effect of treatment.

### Pharmacokinetic analysis of i.m. obiltoxaximab.

PK parameters of obiltoxaximab in cynomolgus macaques following a single i.m. injection were evaluated in the preexposure study and postexposure studies PEP 2 and PEP 3 (Table 3). The maximum concentration of obiltoxaximab in serum (*C*_max_) and the area under the concentration-time curve from time zero extrapolated to infinity (AUC_0–∞_) increased approximately dose proportionally after i.m. administration of 8 to 16 mg/kg. Mean i.m. time to maximum concentration (*T*_max_) values ranged from approximately 0.5 to 3 days, with an overall average of approximately 1 day across studies, which is generally before anthrax-infected monkeys have transitioned to advanced systemic disease, even accounting for the 18- to 36-h postchallenge dose administration. Mean half-life (*t*_1/2_) values ranged from approximately 5 to 12 days, respectively, across studies. In study PEP 2, key PK parameters (*t*_1/2_, apparent clearance after a nonvascular dose [CL/F], and apparent volume of distribution after a nonvascular dose [*V_z_*/F]) all were comparable after a dose of 8 or 16 mg/kg. In all studies, the time of dosing had no effect on PK, suggesting that severity of disease at the time of i.m. dosing had little impact.

**TABLE 3:** Pharmacokinetics of intramuscular obiltoxaximab^*c*^

Study and dose (mg/kg)^*a*^	Postchallenge dosing schedule	*C*_max_ (μg/ml)	*T*_max_ (days)	AUC_(0–∞)_ (μg/day/ml)	*t*_1/2_ (days)	CL/F (ml/day/kg)	*V_z_*/F (ml/kg)
PEP 2							
8	18 h	80.8	1.0	1,030	7.50	7.76	84.0
8	24 h	86.5	1.0	788	5.20	10.2	76.2
8^*b*^	36 h	60.8	1.0				
16	18 h	165	1.0	2,120	8.44	7.54	91.9
16	24 h	119	1.0	1,990	9.85	8.03	114
16	36 h	140	0.50	1,600	8.23	10.0	119
PEP 3							
16	24 h	142	1.0	2,190	10.4	7.00	110
16	36 h	132	0.50	1,540	7.09	10.0	106
16	48 h	104	3.0	2,040	12.6	8.00	143
Preexp							
16	Day −3	152	1.6	1,940	9.95	8.55	117
16	Day −2	156	1.2	1,850	10.0	8.93	126
16	Day −1	149	1.8	1,990	9.23	8.26	108

a Animals were challenged with a target dose of 200 LD_50_ equivalents of B. anthracis (Ames strain) spores. Due to mortality following challenge, PK parameters were based on composite mean concentrations from all animals per time point per dose group (male and female values combined), analyzed as one profile.

b All animals succumbed to anthrax and obiltoxaximab; therefore, concentration data were incomplete.

c Values are combined-sex means.

## DISCUSSION

Inhalational anthrax remains a serious bioterrorist threat with a continuing need for the development of effective therapeutic strategies for prevention and treatment (22). Obiltoxaximab was recently approved under FDA's Animal Rule for treatment of inhalational anthrax in combination with antimicrobial drugs and for prophylaxis of inhalational anthrax when alternative therapies are not available. The approved therapeutic dose of obiltoxaximab is 16 mg/kg administered intravenously. Here, we present the outcomes of pre- and postexposure prophylaxis studies with obiltoxaximab in animals that formed the basis of approval for the prophylaxis indication.

A single dose of obiltoxaximab at 16 mg/kg i.m. was effective in preventing development of inhalational anthrax when given prophylactically 1 to 3 days before spore exposure or when given after the exposure but prior to the development of bacteremia. A range of treatment times (9 to 48 h postchallenge) allowed us to comprehensively investigate postexposure prophylactic effectiveness and to determine the boundaries of the prophylactic window. Treatment administration 9 to 24 h postchallenge and prior to development of systemic disease was considered to be within the boundaries of postexposure prophylaxis as stipulated by the FDA guidance (20). Treatment delay to 36 h when the majority or all animals began to exhibit positive blood bacteremia and PA and when animals may be treated in a trigger-to-treat study (14) led to reduced survival, as predicted for the treatment setting. Because obiltoxaximab was administered at fixed times in our studies and not based on initial manifestation of disease, overall survival was impacted by variations in the extent of systemic disease, with bacteremia reaching high levels in individual animals. When obiltoxaximab administration was delayed to 48 h, all animals had evidence of systemic disease, with a significant proportion of animals exhibiting high pretreatment bacteremia levels (>10^5^ CFU/ml) leading to further reduction in survival. Survival in symptomatic animals was correlated inversely with pretreatment bacteremia levels, comparable to observations reported previously for antibiotics (23).

Improved survival following prophylactic administration of raxibacumab prior to or at the time of spore exposure has been demonstrated (24). The series of studies presented here provide a comprehensive assessment of antitoxin utility in both pre- and postexposure prophylaxis and characterize antitoxin effectiveness relative to the development of symptomatic disease. Beyond survival, results of these studies offer insight into the toxin roles during different stages of disease through examining blood bacteremia and tissue sterilization subsequent to neutralization of toxin. Both i.m. and i.v. routes of administration were explored here. In cynomolgus macaques, when treatment was initiated 24 h postchallenge via the i.v. or i.m. route, survival outcomes were consistently improved. While obiltoxaximab is approved for i.v. administration, the i.m. mode of administration was explored because we reasoned that availability of an i.m. injection option may provide flexibility in mass emergency prophylaxis settings. In addition, because exposures are lower with the intramuscular mode of administration than the intravenous mode, obiltoxaximab administered i.v. is expected to be at least as effective as when given by the intramuscular route. Thus, data for prophylactic efficacy of obiltoxaximab given i.m. also supports the efficacy of i.v. prophylaxis.

It is notable that 3 control macaques survived high spore challenge doses. While testing for circulating anti-PA IgGs was not conducted prior to challenge for the studies described in this work, prescreening for anti-PA IgG was conducted as an exclusion criteria in one obiltoxaximab treatment study (Yamamoto et al., submitted), and one control animal still negative for anti-PA IgG survived the challenge. It should be noted that survival of untreated primates following spore exposure is not unique to obiltoxaximab studies. In a recent study on an intramuscular AVA vaccine regimen in rhesus macaques (25), survival in control groups (saline treated or unvaccinated process control animals) was 13/57 animals following exposure to ∼400 LD_50_ spores. According to the authors of that study, anti-PA IgGs were not detectable until postexposure day 7 in all control survivors, suggesting that reasons other than preexisting antibodies to PA are responsible. The reasons why some of the control primates are able to survive a lethal inhalational anthrax challenge are not known and may include multiple factors, such as immune status prior to challenge and/or genetic polymorphisms in the individual primates. However, we believe that application of rigorous randomization procedures allowed us to generate meaningful and valid survival analyses.

Antibiotic treatment early after spore challenge and prior to establishment of systemic disease can lead to persistence of spores and disease development following antibiotic cessation (26, 27, 28), necessitating a long duration of antibiotic prophylaxis (29). There was no incidence of disease reemergence in our studies during the 28- to 56-day follow-up when obiltoxaximab was given as a single dose pre- or postexposure. The earliest postexposure treatment time was 9 h in the rabbit study, a time when spore germination is beginning to commence (30). The low (33%) survival rate in the antibiotic monotherapy arm of the study was expected because of early discontinuation of the antibiotic regimen. In a recent study by Ionin et al., a comparable survival rate of 23% in NZW rabbits was observed after antibiotic treatment discontinuation (31). Survival in obiltoxaximab-treated animals was 89% to 100%, suggesting that a single dose of obiltoxaximab can provide lasting protection and may provide added benefit to antimicrobials during postexposure prophylaxis. Lack of disease reemergence following obiltoxaximab pre- and early postexposure prophylaxis may be due to the longer half-life of circulating antibodies compared to antibiotics. However, the study duration (28 to 56 days) extended beyond the obiltoxaximab half-life, raising the possibility that challenged and treated animals have developed protective endogenous immunity and/or spores had been eliminated. Although development of endogenous immune responses to B. anthracis was not measured in survivors of our studies, development of anti-PA IgG responses in spore-challenged nonhuman primates has been documented (25).

Overall, we demonstrate that a single 16 mg/kg i.m. dose of obiltoxaximab administered ≤24 h postexposure prevented PA toxemia and bacteremia and significantly improved survival in two animal species expected to react with a response predictive for humans. An 8 mg/kg i.m. obiltoxaximab dose administered up to and including 24 h postchallenge also showed statistically significant improvements in survival compared to the placebo. These results agree well with the established role of PA in enabling bacterial dissemination to the periphery and establishment of systemic infection (32, 33) and support the clinical indication of obiltoxaximab use in prophylaxis.

The survival benefits were time dependent, with higher survival observed with earlier treatment after spore exposure, and were persistent, with no delayed occurrence of inhalational anthrax through the end of the study. Furthermore, added benefit was observed when obiltoxaximab was coadministered with antibiotics in the early postexposure prophylaxis setting in rabbits. Results of the animal efficacy studies presented here support the utility of antitoxin administration in conjunction with standard antimicrobial regimens during inhalational anthrax pre- or postexposure prophylaxis, particularly under conditions when antibiotic noncompliance or antimicrobial resistance is anticipated.
